# Lung Damage Lingers after 9/11

**DOI:** 10.1289/ehp.118-a245

**Published:** 2010-06

**Authors:** Adrian Burton

**Affiliations:** **Adrian Burton** is a biologist living in Spain who also writes regularly for *The Lancet Oncology*, The Lancet Neurology, and Frontiers in Ecology and the Environment

Firefighters and emergency medical services (EMS) workers exposed on 11 September 2001 and in the months thereafter to pulverized building dust from the World Trade Center suffered significant reductions in their lung function—losses that persist 7 years on, according to new research.^1^ “Lost lung function in most firefighters who inhale smoke, even from a chemical fire, normally recovers quite quickly,” explains study leader David Prezant of Albert Einstein College of Medicine. “Surprisingly, our research shows that the firefighters and EMS workers exposed on 9/11 and thereafter have enjoyed no such recovery.”

The researchers examined the FEV_1_ of 12,781 firefighters and EMS workers for whom data were available before and several years after 9/11; every 12–18 months the personnel of these corps undergo routine health assessments that include this measurement. FEV_1_ refers to the maximum volume of air that can be blown out in 1 second; it is one of the primary markers of lung function.

Exposure was estimated by analyzing when subjects first arrived at Ground Zero on 9/11 itself and for how long they were present in the months thereafter. The subjects were also subdivided into those who never smoked, always smoked, or smoked only after 9/11. The authors predicted the FEV_1_ for each subject (adjusted for age-related decline, gender, height and race) for each 6-month period from 12 March 2000 to 11 September 2008, then determined how each actual FEV_1_ measurements compared with the predicted values.

A persistent reduction in lung function was seen in all subgroups. Even among never-smokers, large and significant mean reductions in FEV_1_ occurred over the first year, with greater reductions among firefighters than EMS workers. Over the next 6 years, FEV_1_ failed to ever demonstrate significant recovery.

The results showed that of all the firefighters, those who entered Ground Zero the morning of 9/11 fared the worst. Paul Lioy, an expert on the World Trade Center dust who was not involved in this study, explains these first responders encountered very high levels of a complex mixture comprising glass fibers (from disintegrated windows), high-pH cement particles, unquantified gases, and many other constituents. “It was a sequence of exposures that would depend on the time you were there initially, the time you spent there, and whether you were wearing respiratory protection,” he says.

After 7 years, however, time of arrival did not appear to influence eventual loss of lung function. According to the study authors, this suggests “although the intensity of the initial exposure may have been the critical determinant of acute inflammation and early reductions in lung function, the long-term course was more related to the population that was exposed than to the exposure.”

“The massive dust concentrations to which these people were acutely exposed has produced significant reduction in lung function, predominantly due to airway inflammation resulting in obstructive airways disease—asthma, chronic bronchitis, bronchiolitis,” explains Prezant. “For many, the lungs seem unable to recover from the inflammation caused.”

“No other situation has involved this type of dust in such huge, acute exposures,” remarks Omar Usmani of the National Heart and Lung Institute, Imperial College London & Royal Brompton Hospital. “Such loss of respiratory function would not be expected in people living in even very highly polluted places; the pollutants are different, and the exposure is chronic rather than massively acute. [Even] rescue workers attending urban earthquake disasters would typically not be similarly exposed. What this research shows is how much more prepared and vigilant we need to be in assessing, managing, and protecting the respiratory function of rescue workers exposed to the most extreme situations.”
